# Perinatal Famine Exposure and Young-Onset Cancer—Lessons from China Health and Nutrition Survey

**DOI:** 10.3390/cancers16142537

**Published:** 2024-07-14

**Authors:** Aidi Shuai, Shahid Ullah, Yongfu Yu, Stephen J. Pandol, Savio George Barreto

**Affiliations:** 1 College of Medicine and Public Health, Flinders University, Adelaide, SA 5042, Australia; eddy.shuai@flinders.edu.au; 2 School of Public Health, Shanghai Medical College, Fudan University, Shanghai 200031, China; yu@fudan.edu.cn; 3 Division of Digestive and Liver Diseases, Cedars-Sinai Medical Center, Los Angeles, CA 90048, USA; stephen.pandol@cshs.org; 4 Division of Surgery and Perioperative Medicine, Flinders Medical Centre, Bedford Park, Adelaide, SA 5042, Australia

**Keywords:** young-onset cancer, famine, perinatal, cancer

## Abstract

**Simple Summary:**

The PELICan hypothesis speaks to the role of perinatal and early life stressors (including malnutrition) in the causation of young-onset cancers. To date, there is no evidence correlating perinatal malnutrition and the risk of young-onset cancer. The Great Famine of China was a significant event in human history. The present study compared the incidence of young-onset cancers in individuals born during and just after the famine to those born prior to the famine. Perinatal exposure to famine, especially in females, was associated with a higher risk of young-onset cancer. This was particularly evident for young-onset genitourinary cancers.

**Abstract:**

**Background/Objectives:** Perinatal exposure to malnutrition has been hypothesised to influence the development of young-onset cancer (≤50 years of age). This study aimed to determine if perinatal malnutrition in individuals exposed to the Great Famine of China increased their risk of developing young-onset cancer compared to other individuals born prior to the famine. **Subjects/Methods:** This cross-sectional study involved 7272 participants from the China Health and Nutrition Survey who were classified into four groups based on birth year: participants born between 1953 and 1955 (before the famine) were designated as the pre-famine group (unexposed); the remainder formed perinatal exposure groups comprised of those exposed during the famine (1959–1961), those exposed in the early post-famine period (1962–1964), and those exposed in the late post-famine period (1965–1967). Multivariable adjusted log-binomial regression models were used to calculate the RR and 95% CI of young-onset cancer (including genitourinary cancer) across four groups. **Results:** Perinatal exposure to early post-famine (RR 2.08; 95%CI 1.04, 4.34; *p* = 0.043) and the female sex (RR 15.6, 95%CI 4.54, 60.3; *p* < 0.001) were noted to have a significantly increased risk of young-onset cancer. In addition, the early (RR 13.8; 95%CI 2.68, 253; *p* = 0.012) and late post-famine (RR 12.3; 95%CI 2.16, 231; *p* = 0.020) cohorts demonstrated a significantly increased risk of young-onset genitourinary cancer. The latter was accompanied by an increased risk of hypertension (RR 3.30; 95%CI 1.28, 7.87; *p* = 0.009). **Conclusions:** Perinatal exposure to famine, especially in females, was associated with a higher risk of young-onset cancer. This was particularly evident for young-onset genitourinary cancers. These findings highlight the potential long-term impact of perinatal malnutrition on young-onset carcinogenesis.

## 1. Introduction

Young-onset cancer, a distinct and real entity affecting individuals 50 years old or younger [1,2,3,4,5], is on the rise globally [1,2,3,6,7,8,9]. The most commonly reported organ subsites affected include the colon and rectum [1,9], stomach [1,9], pancreas [4,6], ovaries [5], breasts [10], and oesophagus [11]. The causative factors for young-onset carcinogenesis remain uncertain. Environmental factors have been implicated to play a role [12] based on an increased association noted with these cancers compared to people unaffected by the disease. 

In 2020, Barreto proposed that perinatal and early stressors may be responsible for young-onset carcinogenesis [13]. Together with Professor Pandol, they presented what they referred to as the PELICan (Perinatal and Early Life Influences on Cancer) hypothesis, substantiated with available evidence from literature suggesting that the risk of developing young-onset cancer begins in the perinatal period due to exposure to stressors (including maternal malnutrition, smoking, or alcohol consumption) during foetal life. They hypothesised that the stressors induce epigenomic events intended to assist the foetus in coping with and/or adapting to these stressors [13]. When an individual is exposed to the same stressors early in life, it may reactivate these ‘responses designed to be protective’. However, this reactivation can ultimately lead to a loss of regulation at a metabolic and/or genetic level, culminating in neoplastic evolution [13]. The PELICan hypothesis, drawing on the sentinel work of Barker [14], Knudson [15], and Lahouel [16], remains to be proven. 

The Great Famine of China of 1959 to 1961 was a natural disaster affecting almost the entire Chinese population. It has been widely used for exploring the impact of famine on health outcomes [17,18,19,20,21]. In the present context, it presented a unique opportunity to determine if perinatal exposure to famine was associated with the risk of development of young-onset cancer. To date, no such study has explored this. Hence, the present study aimed to determine if perinatal malnutrition in individuals exposed to the Great Famine of China increased their risk of developing young-onset cancer, compared to those individuals born prior to the famine.

## 2. Methods

### 2.1. Study Population

We performed a cross-sectional analysis of data from the China Health and Nutrition Survey (CHNS), which is an ongoing prospective household-based cohort study in China [22,23,24]. This CHNS study was initiated in 1989, with ten rounds of surveys (1989, 1991, 1993, 1997, 2000, 2004, 2006, 2009, 2011 and 2015), and included a diverse sample of more than 30,000 participants in 7200 households in nine provinces: Shandong, Heilongjiang, Jiangsu, Liaoning, Hunan, Henan, Hubei, Guizhou, and Guangxi [22,23,24]; these provinces vary substantially in geography, public resources, economic development, and health indicators. A multi-stage, random cluster sampling strategy was used to select the sample in both urban and rural areas in each province, with all data being collected via in-person interviews [22,23,24]. Counties in selected provinces were stratified by income level, and four counties with different income levels were randomly selected from each province using a weighted sampling scheme [22,23,24]. Thus, a wide-ranging set of health, nutritional, and demographic indicators are available across the time frame, contributing to the strength of the data source. Cancer information was collected in 2011 and 2015. The methodological details about this study design are available elsewhere [22,23,24]. Ethical approval of CHNS was obtained from all participants [24]. The survey was approved by the institutional review committees [24].

**Figure 1:**
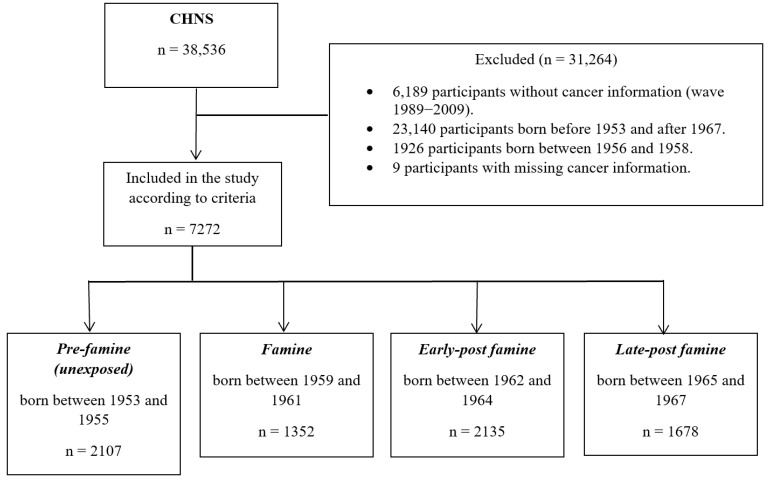
Flowchart of this study population and assessment of exposure (abbreviations: CHNS—China Health and Nutrition Survey).

A total of 7272 eligible participants from round 2011 and 2015 in CHNS were included. Participants born between 1953 and 1955 and between 1959 and 1967 (*n* = 7281) were included. The period from 1956 to 1958 (*n* = 1926) was excluded from the analysis to minimise misclassification, since we were unable to conclusively determine if regions within China had already begun to experience famine in varying proportions in the lead-up to the Great Famine. Nine participants with missing cancer information were excluded. The flowchart of eligible participants is shown in Figure 1.

The effect size reported in previous studies investigating the relationship between perinatal exposure to famine and cancer was examined. In a previous study by Zhang et al. [25] using the CHNS dataset, overall cancer event rates of 0.013 and 0.029 were noted amongst those individuals unexposed and exposed to perinatal famine (hazard ratio/HR:2.11, 95% CI: 1.77, 2.52). Based on these event rates and the HRs from that study, we determined that an overall sample size of 3635 participants (1817 in the unexposed group and 1818 in the exposed group) would achieve 90% power at a 0.05 level of significance, with an equivalence bound of 2.11 and an HR of 1.00. The number of events required to achieve this power was 77.7. It was anticipated that the proportion of participants in which the event was observed during this study was 0.013 for the unexposed group and 0.029 for the exposed group. These results assumed that the HR was constant throughout this study. The sample size in the present study was limited by the availability of data within the CHNS. While the overall number falls short of the anticipated sample size, we believed it served its purpose in addressing the research question being posited. Power Analysis and Sample Size (PASS) software program version 11.0 was used to estimate the sample size [26,27].

### 2.2. Assessment of Perinatal Famine Exposure

The Great Famine of China affected almost the entire country. Thus, the year of birth was used as the most common method to define famine exposure in previous studies [19,25]. In the present study, participants were classified into famine-exposed and unexposed groups based on their perinatal exposure to the famine, i.e., if they were born in those years. Participants (n = 2107) born before the famine (1953–1955) were classified as the unexposed group (or reference group), named the pre-famine group. The cohorts were classified in 3-year intervals owing to the famine lasting for 3 years.

### 2.3. Assessment of Outcome

The primary outcome of this study was the young-onset cancer rate, determined as the number of young-onset cancer cases divided by survey participants. The secondary outcome was the rate of prevalence of the various affected organ subsites of young-onset cancer. Cancer information, including cancer diagnosis, site of cancer, and age at cancer diagnosis, were self-reported and recorded in the CHNS questionnaire section from 2011 to 2015, though the survey was initiated in 1989. Young-onset cancer was ascertained if a participant answered “Yes” and “50 years old or prior” to the questions: “Has a doctor ever given you a diagnose of cancer?” and “How old were you when you first diagnosed with cancer?”, respectively. Thereafter, based on the participant’s answer to the question “What type of cancer you suffer from?”, the cancer was further sub-classified into gastrointestinal cancer (hepatic\stomach\oesophageal\colon), genitourinary cancer (testes\prostate\cervical\uterine), skin cancer (skin\melanoma), breast cancer, young-onset lung cancer, and brain cancer.

### 2.4. Assessment of Covariates

Potential risk factors for young-onset cancer were adjusted for in the analysis phase to reduce the confounding bias [3,13,14,28]. Sex, birth area, physical examination results, health-related behaviour data, and health history were collected via a structured questionnaire. The Great Famine of China affected all provinces in mainland China. However, rural areas suffered more than urban areas due to the preferential supply of food to cities [29]. Hence, we also adjusted for area. The individual’s weight and height were recorded in the physical examination survey [24]. Based on the World Health Organization’s (WHO) criteria for body mass index (BMI), BMI ≤ 18.5 was categorised as underweight, BMI between 18.5–24.9 as normal, BMI of 25–29.9 as overweight, BMI ≥ 30 as obesity. Health history covariates included hypertension and diabetes [30]. Health-related behaviour covariates included smoking status and alcohol consumption [24].

### 2.5. Statistical Analysis

The baseline characteristics of the included participants and perinatal famine exposure groups were compared using the Kruskal–Wallis H test for continuous indicators and chi-square tests for categorical variables. Unadjusted and multivariable adjusted log-binomial regression models were applied to explore the association between perinatal famine exposure and risk of young-onset cancer and young-onset genitourinary cancer. Baseline characteristics (age, sex, BMI, and area), health-related behaviours (smoke and alcohol consumption) and presence of diabetes and hypertension were used to build the multivariable adjusted model. The log-binomial regression model result was expressed as relative risk (RR). Area under the curve (AUC) was used to evaluate model diagnostics and goodness of fit. A two-sided *p*-value of <0.05 level was considered to be statistically significant. All statistical analyses were performed using R statistical software, version 4.2.3 (Vienna, Austria) [31]. The *glm* function with a binomial family log link was used to perform the log-binomial regression model.

## 3. Results

### 3.1. Baseline Characteristics (Table 1)

Among the 7272 participants, 2017 (29.0%) were unexposed to famine in the perinatal period, whilst 1352 (18.6%), 2135 (29.4%) and 1678 (23.1%) were classified as exposed under the pre-specified groups, as follows: famine exposure, early, and late post-famine exposure, respectively. A total of 3838 (52.8%) and 4429 (60.9%) participants were reported to be female and located in a rural area, respectively. The median age of participants was 52 years (interquartile range/IQR 48–56 years). While all participants in the pre-famine group were over 50 years, all participants in late post-famine group < 50 years of age. Compared with the pre-famine group, the famine group was more likely to be from urban area and more likely to have reported the use of alcohol later in life (*p* < 0.01). Furthermore, the early post-famine group participants were more likely to be classified as overweight or obese in later life (*p* < 0.01). The famine, early, and late post-famine exposure groups were less likely to have hypertension and diabetes (*p* < 0.01).

### 3.2. Outcomes (Table 2)

The overall young-onset cancer rate in our study population was 0.73% (53 cases), with rates of 0.57%, 0.59%, 1.08%, and 0.60% in the pre-famine, famine, early-post famine, and late post-famine groups, respectively. Young-onset genitourinary cancer was the most prevalent young-onset cancer sub-type (rate of 0.32%; n = 23 cases). According to exposure, the prevalence rates of young-onset genitourinary cancer were 0.05%, 0.30%, 0.52%, and 0.42% in the pre-famine, famine, early post-famine and late post-famine groups, respectively.

#### 3.2.1. Young-Onset Cancer (Figure 2 and Supplementary Table S1)

After adjustment for sex, area, alcohol consumption, smoking, BMI, hypertension, and diabetes, the early post-famine group had a significantly higher risk of developing young-onset cancer (RR 2.08; 95% CI 1.04, 4.34; *p* = 0.043). However, no relation was observed in the early post-famine and late post-famine groups. Moreover, a highly significant increased risk of young-onset cancer was observed in the female participants (RR 15.6, 95% CI 4.54, 60.3; *p* < 0.001) who experienced perinatal exposure to famine. Additionally, no significant association was observed between area, alcohol consumption, smoking, BMI category, hypertension, and diabetes on young-onset cancer.

#### 3.2.2. Young-Onset Genitourinary Cancer (Figure 3 and Supplementary Table S2)

After adjustment for alcohol consumption, smoking, hypertension, and diabetes, the early post-famine (RR 13.8; 95% CI 2.68, 253; *p* = 0.012) and late post-famine exposure groups (RR 12.3; 95% CI 2.16, 231; *p* = 0.020) were noted to be at a significantly increased risk of developing young-onset genitourinary cancer compared to the pre-famine group. Moreover, participants with perinatal exposure to famine who developed hypertension were noted to have significantly increased risks of young-onset genitourinary cancer compared to those without hypertension (RR 3.30; 95% CI 1.28, 7.87; *p* = 0.009). No significant association was observed between alcohol consumption, smoking, and diabetes on young-onset genitourinary cancer amongst all participants.

## 4. Discussion 

The findings from this study confirm that perinatal exposure in the early post-famine period resulted in a significantly increased risk of young-onset cancers. This risk was significantly higher amongst females. The early post-famine group was also noted to a have a significantly higher rate of alcohol consumption and overweight or obesity in later life compared to the pre-famine cohort (*p* < 0.001). Of all the reported cancers in this study, perinatal exposure in the early post-famine and late post-famine periods resulted in a high risk of young-onset genitourinary cancer that was accompanied by a higher co-existence with hypertension. 

While the relationship between perinatal exposure to the Great Famine of China and an increased risk of cancer, in general, has been previously reported by Zhang et al. [25] and Xie et al. [32], the effect on young-onset carcinogenesis per se has not been determined before. In the study by Zhang et al. [25], perinatal exposure to the Great Famine of China increased the risk (HR: 2.11, 95% CI: 1.77, 2.52) of cancer in adulthood compared to those unexposed to famine. The cancers demonstrating this trend included colorectal, breast (female), lung, stomach, and liver. In the study by Xie et al. [32], the slightly increased overall crude incidence of in situ and invasive breast cancer was reported in women conceived, or born, during the Great Famine of China compared with those women born before (1955–1958) and after (1963–1966). 

We found a highly significant interaction between sex and perinatal famine exposure in relation to young-onset cancer risk. When we ran the models separately for male and female participants, the risk of young-onset cancer in the female group, especially, turned out to be identical to the overall model risk. Thus, we can infer that the elevated risk of cancer related to malnutrition has a predilection towards the female sex. The difference in the incidence of a variety of cancers between the male and female participants may be attributed to genetic and molecular disparities and varying levels of sex hormones [33]. In the study by Zhang et al. [25], females in early childhood (0.1–5.9 years), who were exposed to the famine, were noted to have a higher risk of lung, colorectal, liver, and breast cancer compared to men. 

We observed a significant likelihood of developing young-onset genitourinary cancer in individuals who were perinatally exposed to the famine in the early post-famine period. Moreover, this trend was accompanied by a higher prevalence of hypertension. A study based on the analysis of the Dutch Famine (1944–1945) reported a higher risk of overall cancer in females severely exposed to the famine compared to those who were unexposed [34], whilst another study, based on the same cohort, found that perinatal exposure to the famine amongst females resulted in a higher likelihood of developing breast cancer compared to those who were unexposed [35]. Additionally, the females displayed a natural inclination towards increased vulnerability to the effects of perinatal famine exposure. This heightened susceptibility to the damaging effects of perinatal stressors amongst females is not limited to cancer alone but has also been reported to result in elevated risks of being overweight or obese [36,37], as well as being afflicted with type 2 diabetes [21], metabolic syndrome [38], visceral adipose dysfunction [39], dyslipidaemia [40], and hypertension [17] in later life. Many animal models have provided compelling evidence, indicating that nutrition in early life can have long-lasting effects that span across several generations [41,42,43,44]. Thus, the well-being of offspring is significantly influenced by the nutrition conditions of both parents [41,42,43,44]. 

The potential mechanisms that link perinatal exposures and young-onset cancer are likely complicated. The PELICan hypothesis [13,45] highlights the significance of perinatal malnutrition, both over- and under-nutrition, as stressors. Malnutrition, whether encountered in individuals living with obesity, or more commonly perceived as undernutrition (a direct effect of famine), is known to negatively impact fertility [46,47,48]. Mineral and micronutrient deficiency and inadequate gestational nutrition impact the early physical and neurological growth and development of the child [49]. The long-term effects of these factors and the accompanying biochemical mechanisms that could play a role in carcinogenesis in later life have not been studied. Through the PELICan hypothesis, we have hypothesised the importance of similar stressors in later life to complement these early insults/stressors. We hope that the underlying mechanisms will be investigated and clarified soon. The physiological imbalance that accompanies a sudden change in nutritional status, though, is striking. There is evidence to suggest that children born to mothers who were pregnant at the time of the Chinese Great Leap Forward famine were prone to overnutrition [50] and metabolic syndrome [51], likely owing to adaptive lipogenesis [52]. The Predictive Adaptive Response (PAR) hypothesis by Gluckman and Hanson [53] speaks to a form of developmental plasticity, in which cues received in early life influence the development of a phenotype that is normally adapted to the environmental conditions of later life. This concept is extremely relevant when explaining the findings in the present study. Metabolic dysregulation [54] and obesity [55] have also been reported to be associated with risks of young-onset colorectal cancer. Although not clearly documented as an adolescent exposure, the early post-famine cohort had a significantly increased documentation of another stressor listed in the PELICan hypothesis [13,45], namely, alcohol consumption. Another important consideration is that the potency of dietary carcinogens is increased under conditions of malnutrition, specifically due to a deficiency in protective factors, such as those available from fruits, vegetables, and fibre [56]. 

We wish to acknowledge the limitations of this study. Firstly, the overall number of young-onset cancer cases was low (53, compared to 77 determined by the sample size estimation). While this precluded a more meaningful analysis based on organ subsites beyond the genitourinary tract, the significant findings noted in this study support the hypothesis and rationale for the investigation. Secondly, all health information in this study was self-reported. As a result, the accuracy and reliability of the data may be influenced by self-reporting biases and recall errors. The lack of reliable imaging modalities at the time may have potentially reduced the number of gastrointestinal and brain cancers, since the symptoms of these cancers could be mistakenly attributed to other gastrointestinal and neurological diseases. Thirdly, caution should be exercised due to the possibility of survivorship bias, which may introduce skewed or misleading results. In the CHNS, all cancer-related information was collected from participants who remained in the study between waves 2011 and 2015. It is important to acknowledge that participants may have dropped out during this period due to factors related to famine or young-onset cancer, which could potentially impact the representativeness of the sample and introduce bias. The lack of available data precluded us from controlling for nutrition and physical activity in the intervening years between exposure and outcome. Despite the aforementioned limitations, it is also important to note that the CHNS utilised a multi-stage, random cluster sampling strategy, which ensured that the participants represented a diverse range of socioeconomic statuses and other relevant health, nutritional, and demographic indicators in China. Moreover, the availability of longitudinal data across this study period contributed to the strength and reliability of the data source. Finally, similar to all major famines, there is a lack of accuracy on the actual extent of the famine, with limited analyses of the nutritional and economic effects on the population post-famine [57,58]. 

The results of this study can also be translated to the clinical setting. Acute changes in nutritional status over a prolonged period of time may occur in medicine in the setting of rapid weight loss that accompanies bariatric surgery. One of the benefits following bariatric surgery or even the use of glucagon-like peptide-1 (GLP-1) receptor agonist-induced loss of weight is an improvement in fertility [59,60]. Acknowledging the deleterious effects of these acute nutritional changes (including micronutrient insufficiencies) on pregnancy-related outcomes has led the American Society for Metabolic and Bariatric Surgery (ASMBS), the American Association of Clinical Endocrinology (AACE), and the Obesity Society (TOS) to publish their clinical guidelines advising females to delay becoming pregnant immediately after weight loss surgery to obviate these risks [61]. The findings from this study raise a novel concern that warrants further investigation into the impact of acute nutritional changes at the time of conception and during the perinatal period on the long-term risk of developing cancer in the foetus.

## 5. Conclusions

Perinatal exposure in the early post-famine period, especially in females, resulted in an increased risk of young-onset cancer. This was particularly evident for young-onset genitourinary cancers. These findings highlight the potential long-term impact of perinatal malnutrition on young-onset carcinogenesis.

## Figures and Tables

**Figure 2:**
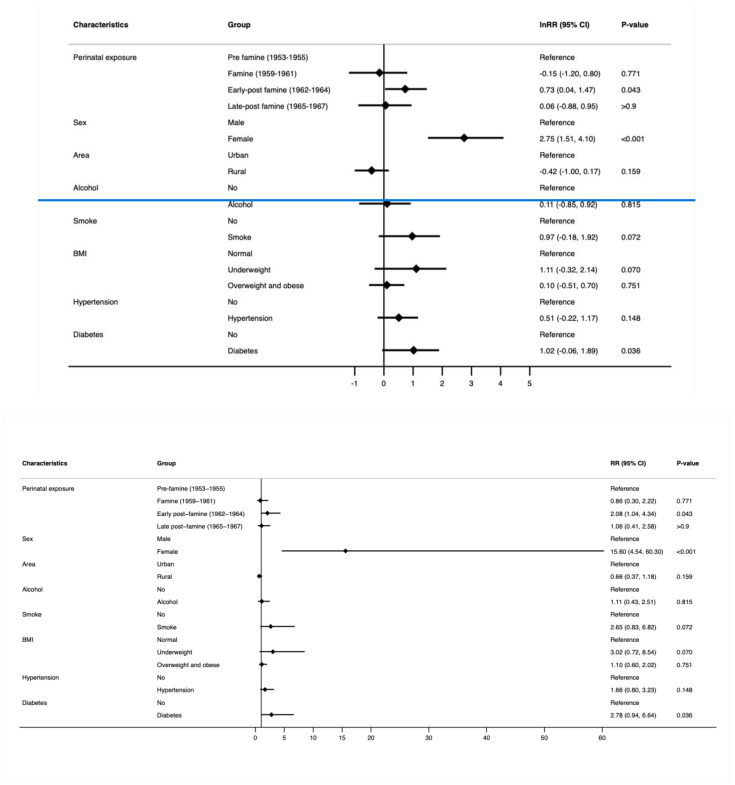
RR and 95% confidence interval (multivariable log-binomial regression model) of young-onset cancer by perinatal famine exposure and confounders. The model was adjusted by sex, area, alcohol consumption, smoking status, BMI, hypertension, and diabetes.

**Figure 3:**
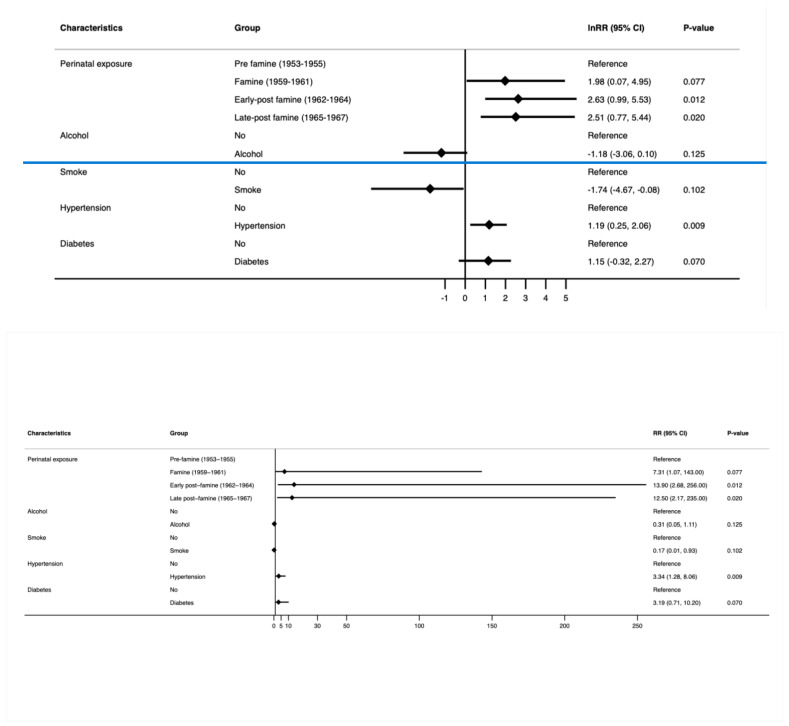
RR and 95% confidence interval (multivariable log-binomial regression model) of young-onset genitourinary cancer by perinatal famine exposure and confounders. The model was adjusted for alcohol consumption, smoking status, hypertension, and diabetes.

**Table 1:** Characteristics of participants across the perinatal famine exposure groups.

Characteristics	Overall (n 7272)	Perinatal Exposure Group				*p*-Value *
		Pre-Famine (n 2107)	Famine (n 1352)	Early Post-Famine (n 2135)	Late Post-Famine (n 1678)	
Age y, median (IQR) †	52.0 (48.0–56.0)	58.0 (57.0,61.0)	53.0 (51.0,55.0)	50.0 (48.0–52.0)	47.0 (45.0–49.0)	<0.001
Age						<0.001
≤50 years	3018 (41.5%)	0 (0.0%)	236 (17.5%)	1104 (51.7%)	1678 (100.0%)	
>50 years	4254 (58.5%)	2107 (100.0%)	1116 (82.5%)	1031 (48.3%)	0 (0.0%)	
Sex						0.267
Male	3434 (47.2%)	967 (45.9%)	645 (47.7%)	1041 (48.8%)	781 (46.5%)	
Female	3838 (52.8%)	1140 (54.1%)	707 (52.3%)	1094 (51.2%)	897 (53.5%)	
Area						<0.001
Urban	2843 (39.1%)	865 (41.1%)	574 (42.5%)	802 (37.6%)	602 (35.9%)	
Rural	4429 (60.9%)	1242 (58.9%)	778 (57.5%)	1333 (62.4%)	1076 (64.1%)	
Alcohol						<0.001
Yes	2422 (33.3%)	620 (29.4%)	471 (34.8%)	775 (36.3%)	556 (33.1%)	
Do not know	135 (1.9%)	29 (1.4%)	23 (1.7%)	47 (2.2%)	36 (2.1%)	
Smoke						0.217
Yes	2190 (30.1%)	643 (30.5%)	428 (31.7%)	647 (30.3%)	472 (28.1%)	
Do not know	136 (1.9%)	30 (1.4%)	23 (1.7%)	46 (2.2%)	37 (2.2%)	
BMI ‡						<0.001
Underweight	168 (2.3%)	66 (3.1%)	27 (2.0%)	36 (1.7%)	39 (2.3%)	
Normal	3923 (53.9%)	1149 (54.5%)	740 (54.7%)	1148 (53.8%)	886 (52.8%)	
Overweight or obese	2840 (39.1%)	823 (39.1%)	525 (38.8%)	848 (39.7%)	644 (38.4%)	
Missing	341 (4.7%)	69 (3.3%)	60 (4.4%)	103 (4.8%)	109 (6.5%)	
Hypertension						<0.001
Yes	1111 (15.3%)	458 (21.7%)	220 (16.3%)	286 (13.4%)	147 (8.8%)	
Do not know	17 (0.2%)	6 (0.3%)	1 (0.1%)	9 (0.4%)	1 (0.1%)	
Diabetes						<0.001
Yes	295 (4.1%)	119 (5.6%)	65 (4.8%)	73 (3.4%)	38 (2.3%)	
Do not know	19 (0.3%)	5 (0.2%)	3 (0.2%)	8 (0.4%)	3 (0.2%)	

Data were presented as n (%) unless stated otherwise, * medians and percentages are compared using Kruskal–Wallis H-test and Pearson’s Chi-squared test, respectively. † IQR 25th–75th percentile, ‡ BMI, body mass index.

**Table 2:** Total young-onset cancer cases and specific site young-onset cancer cases across the perinatal famine exposure groups.

Specific Site-Specific Young-Onset Cancer	Overall (n 7272)	Perinatal Exposure			
		Pre-Famine (n 2107)	Famine (n 1352)	Early Post-Famine (n 2135)	Late Post-Famine (n 1678)
**Young-onset cancer**	53 (0.73%)	12 (0.57%)	8 (0.59%)	23 (1.08%)	10 (0.60%)
**Genitourinary cancer**					
Uterine cancer	22 (0.30%)	1 (0.05%)	4 (0.30%)	11 (0.52%)	6 (0.36%)
Cervical cancer	2 (0.02%)	0 (0.00%)	0 (0.00%)	1 (0.05%)	1 (0.06%)
**Breast cancer**	8 (0.11%)	1 (0.05%)	2 (0.15%)	3 (0.14%)	2 (0.12%)
**Gastrointestinal cancer** Colon cancer	9 (0.12%)	6 (0.28%)	2 (0.15%)	1 (0.05%)	0 (0.00%)
Hepatic cancer	1 (0.01%)	1 (0.05%)	0 (0.00%)	0 (0.00%)	0 (0.00%)
**Brain cancer**	4 (0.06%)	1 (0.05%)	0 (0.00%)	2 (0.09%)	1 (0.06%)
**Lung cancer**	2 (0.03%)	1 (0.05%)	0 (0.00%)	1 (0.05%)	0 (0.00%)
**Other cancer**	9 (0.12%)	4 (0.19%)	0 (0.00%)	5 (0.23%)	0 (0.00%)

## Data Availability

The analysis was performed on data that are publicly available on the website of China Health and Nutrition Survey (China Health and Nutrition Survey—China Health and Nutrition Survey (CHNS) (unc.edu) Access date: 14 March 2023).

## Supplementary Materials

The following supporting information can be downloaded at: https://www.mdpi.com/article/10.3390/cancers16142537/s1, Table S1: RR and 95% Confidence interval (log-binomial regression model) of young-onset cancer by perinatal famine exposure and confounders. Table S2: RR and 95% CI (log-binomial regression model) young-onset genitourinary cancer by perinatal famine exposure and confounders.

## References

[1] Schell D., Ullah S., Brooke-Smith M.E., Hollington P., Yeow M., Karapetis C.S., Watson D.I., Pandol S.J., Roberts C.T., Barreto S.G. (2022). Gastrointestinal Adenocarcinoma Incidence and Survival Trends in South Australia, 1990–2017. Cancers.

[2] Fanny E.R.V., Stella A.V.N., Bardou M., Lansdorp-Vogelaar I., Dinis-Ribeiro M., Bento M.J., Zadnik V., Pellisé M., Esteban L., Kaminski M.F. (2019). Increasing incidence of colorectal cancer in young adults in Europe over the last 25 years. Gut.

[3] Murphy C.C., Singal A.G., Baron J.A., Sandler R.S. (2018). Decrease in Incidence of Young-Onset Colorectal Cancer Before Recent Increase. Gastroenterology.

[4] McWilliams R.R., Maisonneuve P., Bamlet W.R., Petersen G.M., Li D., Risch H.A., Yu H., Fontham E.T.H., Luckett B., Bosetti C. (2016). Risk Factors for Early-Onset and Very-Early-Onset Pancreatic Adenocarcinoma: A Pancreatic Cancer Case-Control Consortium (PanC4) Analysis. Pancreas.

[5] Bernards S.S., Norquist B.M., Harrell M.I., Agnew K.J., Lee M.K., Walsh T., Swisher E.M. (2016). Genetic characterization of early onset ovarian carcinoma. Gynecol. Oncol..

[6] Deng Y. (2017). Rectal Cancer in Asian vs. Western Countries: Why the Variation in Incidence?. Curr. Treat. Options Oncol..

[7] Mohandas K.M., Desai D.C. (1999). Epidemiology of digestive tract cancers in India. V. Large and small bowel. Indian J. Gastroenterol..

[8] American Cancer Society (2020). Special Section: Cancer in Adolescents and Young Adults. Cancer Facts & Figures.

[9] Shepherdson M., Leemaqz S., Singh G., Ryder C., Ullah S., Canuto K., Young J.P., Price T.J., McKinnon R.A., Pandol S.J. (2022). Young-Onset Gastrointestinal Adenocarcinoma Incidence and Survival Trends in the Northern Territory, Australia, with Emphasis on Indigenous Peoples. Cancers.

[10] Chelmow D., Pearlman M.D., Young A., Bozzuto L., Dayaratna S., Jeudy M., Kremer M.E., Scott D.M., O’Hara J.S. (2020). Executive Summary of the Early-Onset Breast Cancer Evidence Review Conference. Obstet. Gynecol..

[11] van Nistelrooij A.M.J., van Marion R., Biermann K., Spaander M.C.W., van Lanschot J.J.B., Wijnhoven B.P.L., Dinjens W.N.M. (2016). Early onset esophageal adenocarcinoma: A distinct molecular entity?. Oncoscience.

[12] Ben-Aharon I., van Laarhoven H.W.M., Fontana E., Obermannova R., Nilsson M., Lordick F. (2023). Early-Onset Cancer in the Gastrointestinal Tract Is on the Rise-Evidence and Implications. Cancer Discov..

[13] Barreto S.G., Pandol S.J. (2021). Young-Onset Carcinogenesis—The Potential Impact of Perinatal and Early Life Metabolic Influences on the Epigenome. Front. Oncol..

[14] Barker D.J. (2007). The origins of the developmental origins theory. J. Intern. Med..

[15] Knudson A.G. (1971). Mutation and cancer: Statistical study of retinoblastoma. Proc. Natl. Acad. Sci. USA.

[16] Lahouel K., Younes L., Danilova L., Giardiello F.M., Hruban R.H., Groopman J., Kinzler K.W., Vogelstein B., Geman D., Tomasetti C. (2020). Revisiting the tumorigenesis timeline with a data-driven generative model. Proc. Natl. Acad. Sci. USA.

[17] Chen H., Nembhard W.N., Stockwell H.G. (2014). Sex-Specific Effects of Fetal Exposure to the 1959–1961 Chinese Famine on Risk of Adult Hypertension. Matern. Child Health J..

[18] Gooch E. (2017). Estimating the Long-Term Impact of the Great Chinese Famine (1959–61) on Modern China. World Dev..

[19] Liu D., Yu D.-M., Zhao L.-Y., Fang H.-Y., Zhang J., Wang J.-Z., Yang Z.-Y., Zhao W.-H. (2019). Exposure to Famine During Early Life and Abdominal Obesity in Adulthood: Findings from the Great Chinese Famine During 1959–1961. Nutrients.

[20] Song S. (2013). Assessing the impact of in utero exposure to famine on fecundity: Evidence from the 1959–61 famine in China. Popul. Stud..

[21] Wang J., Li Y., Han X., Liu B., Hu H., Wang F., Li X., Yang K., Yuan J., Yao P. (2016). Exposure to the Chinese Famine in Childhood Increases Type 2 Diabetes Risk in Adults. J. Nutr..

[22] Zhang B., Zhai F.Y., Du S.F., Popkin B.M. (2014). The China Health and Nutrition Survey, 1989–2011. Obes. Rev..

[23] Popkin B.M., Du S., Zhai F., Zhang B. (2010). Cohort Profile: The China Health and Nutrition Survey—Monitoring and understanding socio-economic and health change in China, 1989–2011. Int. J. Epidemiol..

[24] Prevention, China Center for Disease Control China Health and Nutrition Survey. https://www.cpc.unc.edu/projects/china.

[25] Zhang X., Wang G., Forman M.R., Fu Q., Rogers C.J., Wu S., Gao X. (2021). In utero and childhood exposure to the Great Chinese Famine and risk of cancer in adulthood: The Kailuan Study. Am. J. Clin. Nutr..

[26] Shein-Chung C., Chow S.-C., Shao J., Wang H., Lokhnygina Y. (2017). Sample Size Calculations in Clinical Research.

[27] Schoenfeld D.A. (1983). Sample-Size Formula for the Proportional-Hazards Regression Model. Biometrics.

[28] Barker D.J. (1990). The fetal and infant origins of adult disease. BMJ.

[29] Lin J.Y., Yang D.T. (2000). Food Availability, Entitlements and the Chinese Famine of 1959-61. Econ. J..

[30] World Health Organization (1995). Physical Status: The Use and Interpretation of Anthropometry: Report of a WHO Expert Committee.

[31] R Core Team R: A Language and Environment for Statistical Computing. https://www.R-project.org/.

[32] Xie S.H., Lagergren J. (2017). A possible link between famine exposure in early life and future risk of gastrointestinal cancers: Implications from age-period-cohort analysis. Int. J. Cancer.

[33] Kim H.-I., Lim H., Moon A. (2018). Sex Differences in Cancer: Epidemiology, Genetics and Therapy. Biomol. Ther..

[34] Eriksen K.G., Radford E.J., Silver M.J., Fulford A.J.C., Wegmüller R., Prentice A.M. (2017). Influence of intergenerational in utero parental energy and nutrient restriction on offspring growth in rural Gambia. FASEB J..

[35] Painter R.C., De Rooij S.R., Bossuyt P.M.M., Osmond C., Barker D.J.P., Bleker O.P., Roseboom T.J. (2006). A possible link between prenatal exposure to famine and breast cancer: A preliminary study. Am. J. Hum. Biol..

[36] Saulnier D.D., Brolin K. (2015). A systematic review of the health effects of prenatal exposure to disaster. Int. J. Public Health.

[37] Zhou J., Zhang L., Xuan P., Fan Y., Yang L., Hu C., Bo Q., Wang G., Sheng J., Wang S. (2018). The relationship between famine exposure during early life and body mass index in adulthood: A systematic review and meta-analysis. PLoS ONE.

[38] Ning F., Ren J., Song X., Zhang D., Liu L., Zhang L., Sun J., Zhang D., Pang Z., Qiao Q. (2019). Famine Exposure in Early Life and Risk of Metabolic Syndrome in Adulthood: Comparisons of Different Metabolic Syndrome Definitions. J. Diabetes Res..

[39] Chen C., Zhao L., Ning Z., Li Q., Han B., Cheng J., Chen Y., Nie X., Xia F., Wang N. (2019). Famine exposure in early life is associated with visceral adipose dysfunction in adult females. Eur. J. Nutr..

[40] Wang Z., Li C., Yang Z., Ma J., Zou Z. (2017). Fetal and infant exposure to severe Chinese famine increases the risk of adult dyslipidemia: Results from the China health and retirement longitudinal study. BMC Public Health.

[41] Wei Y., Schatten H., Sun Q.-Y. (2015). Environmental epigenetic inheritance through gametes and implications for human reproduction. Hum. Reprod. Update.

[42] Radford E.J., Ito M., Shi H., Corish J.A., Yamazawa K., Isganaitis E., Seisenberger S., Hore T.A., Reik W., Erkek S. (2014). In utero undernourishment perturbs the adult sperm methylome and intergenerational metabolism. Science.

[43] Carone B.R., Fauquier L., Habib N., Shea J.M., Hart C.E., Li R., Bock C., Li C., Gu H., Zamore P.D. (2010). Paternally Induced Transgenerational Environmental Reprogramming of Metabolic Gene Expression in Mammals. Cell.

[44] Jimenez-Chillaron J.C., Isganaitis E., Patti M.-E., Charalambous M., Gesta S., Pentinat-Pelegrin T., Faucette R.R., Otis J.P., Chow A., Diaz R. (2009). Intergenerational Transmission of Glucose Intolerance and Obesity by In Utero Undernutrition in Mice. Diabetes.

[45] Barreto S.G. (2020). We Asked the Experts: Providing the Road Map to Uncovering the Pathophysiology of Young-Onset Cancer to Guide Treatment and Preventive Strategies. World J. Surg..

[46] Rich-Edwards J.W., Spiegelman D., Garland M., Hertzmark E., Hunter D.J., Colditz G.A., Willett W.C., Wand H., Manson J.E. (2002). Physical Activity, Body Mass Index, and Ovulatory Disorder Infertility. Epidemiology.

[47] Roba K.T., Hassen T.A., Wilfong T., Legese Alemu N., Darsene H., Zewdu G., Negese T., Yifru B., Mohammed E., Raru T.B. (2022). Association of undernutrition and female infertility in East Africa: Finding from multi-country demographic and health surveys. Front. Glob. Women’s Health.

[48] Best D., Avenell A., Bhattacharya S. (2017). How effective are weight-loss interventions for improving fertility in women and men who are overweight or obese? A systematic review and meta-analysis of the evidence. Hum. Reprod. Update.

[49] Farias P.M., Marcelino G., Santana L.F., de Almeida E.B., Guimaraes R.C.A., Pott A., Hiane P.A., Freitas K.C. (2020). Minerals in Pregnancy and Their Impact on Child Growth and Development. Molecules.

[50] Jiang H., Yu Y., Li L., Xu W. (2021). Exposure to the Great Famine in Early Life and the Risk of Obesity in Adulthood: A Report Based on the China Health and Nutrition Survey. Nutrients.

[51] Zheng X., Wang Y., Ren W., Luo R., Zhang S., Zhang J.H., Zeng Q. (2012). Risk of metabolic syndrome in adults exposed to the great Chinese famine during the fetal life and early childhood. Eur. J. Clin. Nutr..

[52] Marcelino H., Veyrat-Durebex C., Rohner-Jeanrenaud F., Dulloo A.G., Summermatter S., Sarafian D., Miles-Chan J., Arsenijevic D., Zani F., Montani J.-P. (2013). A Role for Adipose Tissue De Novo Lipogenesis in Glucose Homeostasis During Catch-up Growth: A Randle Cycle Favoring Fat Storage. Diabetes.

[53] Bateson P., Gluckman P., Hanson M. (2014). The biology of developmental plasticity and the Predictive Adaptive Response hypothesis. J. Physiol..

[54] Chen H., Zheng X., Zong X., Li Z., Li N., Hur J., Fritz C.D.L., Chapman Jr W., Nickel K.B., Tipping A. (2021). Metabolic syndrome, metabolic comorbid conditions and risk of early-onset colorectal cancer. Gut.

[55] Li H., Boakye D., Chen X., Hoffmeister M., Brenner H. (2021). Association of Body Mass Index With Risk of Early-Onset Colorectal Cancer: Systematic Review and Meta-Analysis. Am. J. Gastroenterol..

[56] Lutz W.K. (1999). Carcinogens in the diet vs. overnutrition. Individual dietary habits, malnutrition, and genetic susceptibility modify carcinogenic potency and cancer risk. Mutat. Res..

[57] Chen Y., Zhou L.-A. (2007). The long-term health and economic consequences of the 1959–1961 famine in China. J. Health Econ..

[58] Smil V. (1999). China’s great famine: 40 years later. BMJ.

[59] Guelinckx I., Devlieger R., Vansant G. (2009). Reproductive outcome after bariatric surgery: A critical review. Hum. Reprod. Update.

[60] Pavli P., Triantafyllidou O., Kapantais E., Vlahos N.F., Valsamakis G. (2024). Infertility Improvement after Medical Weight Loss in Women and Men: A Review of the Literature. Int. J. Mol. Sci..

[61] Mechanick J.I., Youdim A., Jones D.B., Garvey W.T., Hurley D.L., McMahon M.M., Heinberg L.J., Kushner R., Adams T.D., Shikora S. (2013). Clinical practice guidelines for the perioperative nutritional, metabolic, and nonsurgical support of the bariatric surgery patient—2013 update: Cosponsored by american association of clinical endocrinologists, The obesity society, and american society for metabolic & bariatric surgery. Obesity.

