# Supplementary material for: Perinatal Famine Exposure and Young-Onset Cancer—Lessons from China Health and Nutrition Survey
Source: Cancers (Basel). 2024 Jul 14;16(14):2537. doi: 10.3390/cancers16142537 (PMC11275197; doi:10.3390/cancers16142537)
Supplement: Supplementary file 1 [file cancers-16-02537-s001.zip › cancers-3092672-supplementary.pdf]

Supplementary Table S1. RR and 95% Confidence interval (log-binomial regression model) of young-onset cancer by perinatal famine exposure and confounders.

	Unadjusted			Multivariable *		
**Perinatal exposure and characteristics**	RR	95% CI	p-value	RR	95% CI	p-value
**Perinatal exposure**						
Pre-famine	1.00			1.00		
Famine	1.04	0.41, 2.51	>0.9	0.86	0.30, 2.22	0.771
Early-post famine	1.89	0.96, 3.92	0.072	2.08	1.04, 4.34	0.043
Late-post famine	1.05	0.44, 2.42	>0.9	1.06	0.41, 2.58	0.900
**Sex**						
Male	1.00			1.00		
Female	8.59	3.77, 24.7	<0.001	15.6	4.54, 60.3	<0.001
**Area**						
Urban	1.00			1.00		
Rural	0.62	0.36, 1.06	0.079	0.66	0.37, 1.18	0.159
**Alcohol**	0.36	0.16, 0.73	0.008	1.11	0.43, 2.51	0.815
**Smoke**	0.36	0.15, 0.75	0.012	2.65	0.83, 6.82	0.072
**BMI**						
Normal	1.00			1.00		
Underweight	2.80	0.67, 7.87	0.089	3.02	0.72, 8.54	0.070
Overweight or obese	1.16	0.64, 2.07	0.60	1.10	0.60, 2.02	0.751
**Hypertension**	1.80	0.93, 3.26	0.065	1.66	0.80, 3.23	0.148
**Diabetes**	3.01	1.16, 6.44	0.010	2.78	0.94, 6.64	0.036

*Multivariable model was adjusted by sex, area, alcohol consumption, smoking status, BMI (body mass index), hypertension and diabetes.

Supplementary Table S2. RR and 95% CI (log-binomial regression model) young-onset genitourinary cancer by perinatal famine exposure and confounders

	Unadjusted			Multivariable *		
**Perinatal exposure and characteristics**	RR	95% CI	p-value	RR	95% CI	p-value
**Perinatal exposure**						
Pre-famine	1.00			1.00		
Famine	6.24	0.92, 122	0.10	7.24	1.07, 142	0.077
Early-post famine	10.9	2.12, 199	0.022	13.8	2.68, 253	0.012
Late-post famine	8.79	1.57, 164	0.042	12.3	2.16, 231	0.020
**Sex**						
Male	1.00			-	-	-
Female	31,646,074	0.00, Inf	>0.9	-	-	-
**Area**						
Urban	1.00			-	-	-
Rural	0.70	0.31, 1.61	0.4	-	-	-
**Alcohol**	0.19	0.03, 0.63	0.023	0.31	0.05, 1.11	0.125
**Smoke**	0.10	0.01, 0.49	0.026	0.17	0.01, 0.93	0.102
**BMI**						
Normal	1.00			-	-	-
Underweight	2.33	0.13, 12.1	0.4	-	-	-
Overweight or obese	1.24	0.49, 3.08	0.6	-	-	-
**Hypertension**	2.95	1.19, 6.78	0.013	3.30	1.28, 7.87	0.009
**Diabetes**	3.53	0.84, 10.2	0.041	3.16	0.73, 9.68	0.070

*Multivariable model was adjusted by alcohol consumption, smoking status, hypertension and diabetes.
